# Appendectomy and appendicitis do not increase colorectal cancer risk: evidence from Mendelian randomization

**DOI:** 10.3389/fonc.2024.1414946

**Published:** 2024-07-22

**Authors:** Wei Wei, Juanhong Wang, Daihua Yu, Wei Liu, Lei Zong

**Affiliations:** ^1^ Department of Pathology, Xi’an No.3 Hospital, the Affiliated Hospital of Northwest University, Xi’an, Shaanxi, China; ^2^ Department of Intensive Care Unit, Xi’an No.3 Hospital, the Affiliated Hospital of Northwest University, Xi’an, Shaanxi, China

**Keywords:** appendectomy, appendicitis, colorectal cancer, Mendelian randomization, risk

## Abstract

**Background:**

Acute appendicitis (AA) is one of the most prevalent acute abdominal diseases and appendectomy is the definitive treatment of appendicitis. However, whether appendicitis and appendectomy cause colorectal cancer (CRC) is controversial. The results of observational studies are contradictory, but randomized controlled trials (RCT) cannot be conducted.

**Methods:**

Data of appendectomy, AA, and CRC were obtained from the IEU Open GWAS project. We selected several Genome-wide association studies (GWAS) summary statistics for CRC: statistics for colon cancer (CC) were obtained from MRC-IEU and Neale lab, respectively; statistics for rectum cancer (RC) were obtained from MRC-IEU and FinnGen, respectively; statistics for CRC were provided by Sakaue S et al. Mendelian randomization (MR) was used to evaluate the causal relationships between exposure and outcomes. Inverse variance weighting (IVW) was the most important analysis method. Meta-analysis was used to summarize the results of IVW to increase the reliability and sensitivity analysis was used to evaluate the robustness of the results.

**Results:**

According to the results of IVW, appendectomy did not increase risk of CC: MRC-IEU (OR:1.009, 95%CI:0.984-1.035, P=0.494), Neale lab (OR:1.016, 95%CI:0.993-1.040, P=0.174); Appendectomy also did not increase risk of RC: MRC-IEU(OR:0.994, 95%CI:0.974-1.014, P=0.538), FinnGen(OR:2.791, 95%CI:0.013-580.763, P=0.706); Appendectomy also did not increase risk of CRC: Sakaue S(OR:1.382, 95%CI:0.301-6.352, P=0.678). Appendicitis did not increase risk of CC: MRC-IEU(OR:1.000, 95%CI:0.999-1.001, P=0.641), Neale lab(OR:1.000, 95%CI:1.000-1.001, P=0.319); Appendicitis also did not increase risk of RC: MRC-IEU(OR:1.000, 95%CI:0.999-1.000, P=0.361), FinnGen(OR:0.903, 95%CI:0.737-1.105, P=0.321); Appendicitis also did not increase risk of CRC: Sakaue S (OR:1.018, 95%CI:0.950-1.091, P=0.609). The results of Meta-analysis also showed appendectomy (P=0.459) and appendicitis (P=0.999) did not increase the risk of CRC.

**Conclusions:**

Appendectomy and appendicitis do not increase the risk of colorectal cancer. More clinical trials are needed in the future to verify the causal relationships.

## 1 Introduction

Acute appendicitis (AA) is one of the most prevalent acute abdominal diseases (1, 2). According to epidemiological data from different countries, lifetime incidence rate of AA can be as high as close to 20% (1–4), and the global incidence rate of AA has increased by more than 60% in the past 30 years (5). Appendicitis is often treated by appendectomy (6, 7), which is the most common emergency abdominal surgery in western countries, with an average of 100 cases per 100,000 person (8). However, many observational studies have revealed that appendectomy may increase the risk of colorectal cancer(CRC): results from Shi et al. showed a 73.0% increase in CRC risk among appendectomy cases throughout 20 years follow-up (SHR:1.73, 95% CI:1.49-2.01, P < 0.001) (9) and results from Chen et al. showed appendectomy was independent risk factors for CRC (OR: 9.10; 95%CI:1.83–50.02, P=0.0055) (10)^]^, AA itself may be a precursor of CRC, which is based on the results of several retrospective studies with follow-up over 5 years: (HR = 4.67; 95% CI: 3.51-6.21, P=0.0011) (11, 12). Other studies have shown that appendectomy can reduce the risk of CRC (HR = 0.90, 95% CI = 0.81‐0.99) (13, 14). Therefore, it is still controversial whether appendicitis and appendectomy are the etiology of(CRC). Due to the limitation of ethics, the relevant randomized controlled trial (RCT) cannot be carried out.

Mendelian randomization (MR) facilitates the study of the causal relationships between exposure factors and outcomes (15, 16). In MR, the single nucleotide polymorphisms (SNPs) from genome-wide association studies (GWASs) were used as instrumental variables (IVs), and the causality was analyzed by IVs instead of exposure factors (15, 16). The time sequence of exposure and outcomes in MR is reasonable, which avoids reverse causality (15, 16). MR is not subject to ethical constraints, and its level of evidence is equivalent to RCT (17). Therefore, this study intends to explore whether appendicitis and appendectomy can cause CRC by MR.

## 2 Materials and methods

### 2.1 Study design

**Figure 1:**
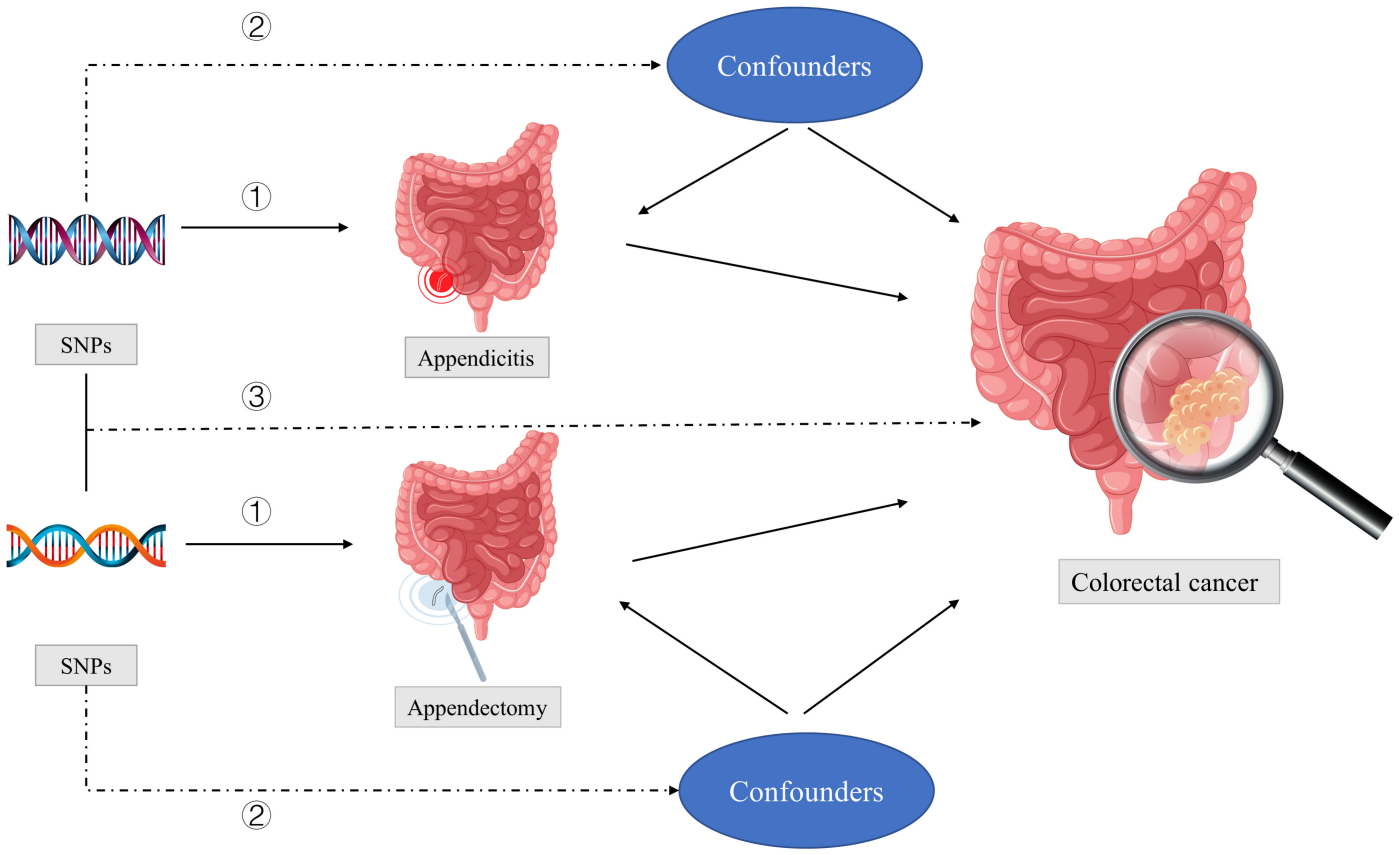
Three key assumptions of this MR study: ① SNPs are strongly associated with appendectomy or appendicitis; ②SNPs are independent of known confounders; ③SNPs only affect colorectal cancer via appendectomy or appendicitis. SNPs, single-nucleotide polymorphisms.

**Figure 1** showed three key assumptions of this MR study: ① SNPs are strongly associated with appendectomy or appendicitis; ②SNPs are independent of known confounders; ③SNPs only affect CRC via appendectomy or appendicitis.

### 2.2 Data sources

The data used in this study were all from the public GWASs and all from Europeans. We obtained the data from the IEU Open GWAS (https://gwas.mrcieu.ac.uk/); GWAS summary statistics for appendicectomy (SNPs= 9,851,867) were obtained from MRC-IEU; GWAS summary statistics for appendicitis (SNPs= 12,243,521) were obtained from UK Biobank; GWAS summary statistics for colon cancer (CC) were obtained from MRC-IEU (SNPs= 9,851,867) and Neale lab (SNPs= 10,833,390) respectively; GWAS summary statistics for rectum cancer (RC) were obtained from MRC-IEU (SNPs= 9,851,867) and FinnGen (SNPs= 16,380,466) respectively; GWAS summary statistics for CRC were obtained from the data reported by Sakaue S et al. (SNPs= 24,182,361). **Supplementary Table 1** showed the baseline characteristics of selected GWAS summary statistics. We deleted SNPs related to confounders and outcomes variables through the PhenoScanner database (http://www.phenoscanner.medschl.cam.ac.uk/). There was no requirement for further ethical clearance because we used publicly available GWAS data.

### 2.3 Selection of SNPs

The selection of SNPs must meet three requirements (18, 19): First, we selected SNPs associated with appendicectomy at the genome-wide significance threshold with p < 5 × 10^−8^ and we adjusted the significance threshold of appendicitis to 1× 10^−5^ to obtain enough SNPs; Second, in order to ensure the independence of selected SNPs, we need to eliminate the linkage disequilibrium (r^2^ > 0.001, clumping window =10,000 kb); Third, we selected strong IVs according to F-statistics (F=β^2^/SE^2^). When F > 10, SNP was considered a strong instrumental variable. Before performing the MR analysis, we also conducted data-harmonization steps, as the effects of an SNP on the exposure and the outcome had to correspond to the same allele.

### 2.4 MR analysis

Inverse variance weighted (IVW)was the most important analysis method. Sensitivity analysis was used to evaluate the robustness of selected SNPs. To increase the reliability of the results, we chose several GWAS summary statistics of outcome variables. We then employed Meta-analysis to compile the IVW method’s results in order to further increase the reliability.

Pleiotropy, MR-Egger and MR-PRESSO were used to evaluate pleiotropy of selected SNPs. The weighted-median method can provide valid estimates if more than 50% of information comes from valid IVs (20); Cochrane’s Q-value was used to evaluate the heterogeneity of SNPs: When there was heterogeneity, random effect model was used, otherwise, common effect model was used. A significance level of P < 0.05 was considered statistically significant. The analysis was performed by “TwoSampleMR” packages in R version 4.1.3 (R Foundation for Statistical Computing, Vienna, Austria).

## 3 Results

### 3.1 Selection of SNPs

Appendectomy: First, after significance test and removal of linkage disequilibrium, there were 18 SNPs related to appendectomy; Then, 4 SNPs related to confounders were excluded (rs34236350, rs2524069, rs224029, rs10849448). **Supplementary Table 2** showed the final SNPs after data-harmonization step.

Appendicitis: After significance testing and removal of linkage disequilibrium, there were 19 SNPs related to appendicitis. **Supplementary Table 2** showed the final SNPs after data-harmonization step.

### 3.2 Results of MR

**Table 1** showed results of MR and **Table 2** showed results of sensitivity analysis.

**Table 1:** Associations between genetically predicted exposure and outcomes using the Mendelian randomization.

Exposure	Outcomes	IVW		MR-egger		Weighted Median	
		OR(95%CI)	P-value	OR(95%CI)	P-value	OR(95%CI)	P-value
Appendicectomy	Colon cancer(MRC-IEU)	1.009(0.984, 1.035)	0.494	0.947(0.894, 1.004)	0.128	1.001(0.976, 1.027)	0.925
Appendicectomy	Colon cancer (Neale lab)	1.016(0.993, 1.040)	0.174	0.967(0.898, 1.041)	0.392	1.008(0.977, 1.041)	0.617
Appendicectomy	Rectum cancer(MRC-IEU)	0.994(0.974, 1.014)	0.538	0.934(0.887, 0.984)	0.051	0.989(0.965, 1.013)	0.358
Appendicectomy	Rectum cancer(FinnGen)	2.791(0.013, 580.763)	0.706	8.951E-02(1.923E-09, 4.167E+06)	0.794	1.753(0.001, 2.090E+03)	0.877
Appendicectomy	Colorectal cancer (Sakaue S)	1.382(0.301, 6.352)	0.678	0.086(0.001, 10.585)	0.340	1.118(0.148, 8.447)	0.914
Appendicitis	Colon cancer(MRC-IEU)	1.000(0.999, 1.001)	0.641	0.999(0.996, 1.002)	0.365	0.999(0.999, 1.000)	0.228
Appendicitis	Colon cancer (Neale lab)	1.000(1.000, 1.001)	0.319	1.001(0.999, 1.003)	0.394	1.000(0.999, 1.001)	0.894
Appendicitis	Rectum cancer(MRC-IEU)	1.000(0.999, 1.000)	0.361	0.998(0.995, 1.000)	0.074	1.000(0.999, 1.001)	0.578
Appendicitis	Rectum cancer(FinnGen)	0.903(0.737, 1.105)	0.321	0.735(0.470, 1.151)	0.197	0.815(0.620, 1.072)	0.144
Appendicitis	Colorectal cancer (Sakaue S)	1.018(0.950, 1.091)	0.609	1.024(0.845, 1.241)	0.811	0.993(0.912, 1.082)	0.879

IVW, inverse-variance weighted; OR, odds ratio; CI, confidence interval.

**Table 2:** Mendelian randomization in sensitivity analyses predicts causal relationships of exposure and outcomes.

Exposure	Outcomes	Pleiotropy		Heterogeneity		MR-PRESSO
		Intercept	P-value	Q	P-value	P-value
Appendicectomy	Colon cancer(MRC-IEU)	4.000E-04	0.074	12.285	0.056	0.090
Appendicectomy	Colon cancer (Neale lab)	3.000E-04	0.196	9.311	0.676	0.759
Appendicectomy	Rectum cancer(MRC-IEU)	4.000E-04	0.057	7.639	0.266	0.254
Appendicectomy	Rectum cancer(FinnGen)	2.100E-02	0.695	13.313	0.347	0.392
Appendicectomy	Colorectal cancer (Sakaue S)	1.700E-02	0.258	7.946	0.789	0.847
Appendicitis	Colon cancer(MRC-IEU)	2.000E-04	0.410	15.126	0.128	0.078
Appendicitis	Colon cancer (Neale lab)	-7.339E-05	0.617	15.821	0.537	0.410
Appendicitis	Rectum cancer(MRC-IEU)	3.000E-04	0.097	8.783	0.553	0.557
Appendicitis	Rectum cancer(FinnGen)	3.360E-02	0.330	22.200	0.177	0.212
Appendicitis	Colorectal cancer (Sakaue S)	-1.000E-03	0.950	22.146	0.179	0.224

OR, odds ratio; CI, confidence interval.

#### 3.2.1 Effect of appendectomy on CRC

**Figure 2:**
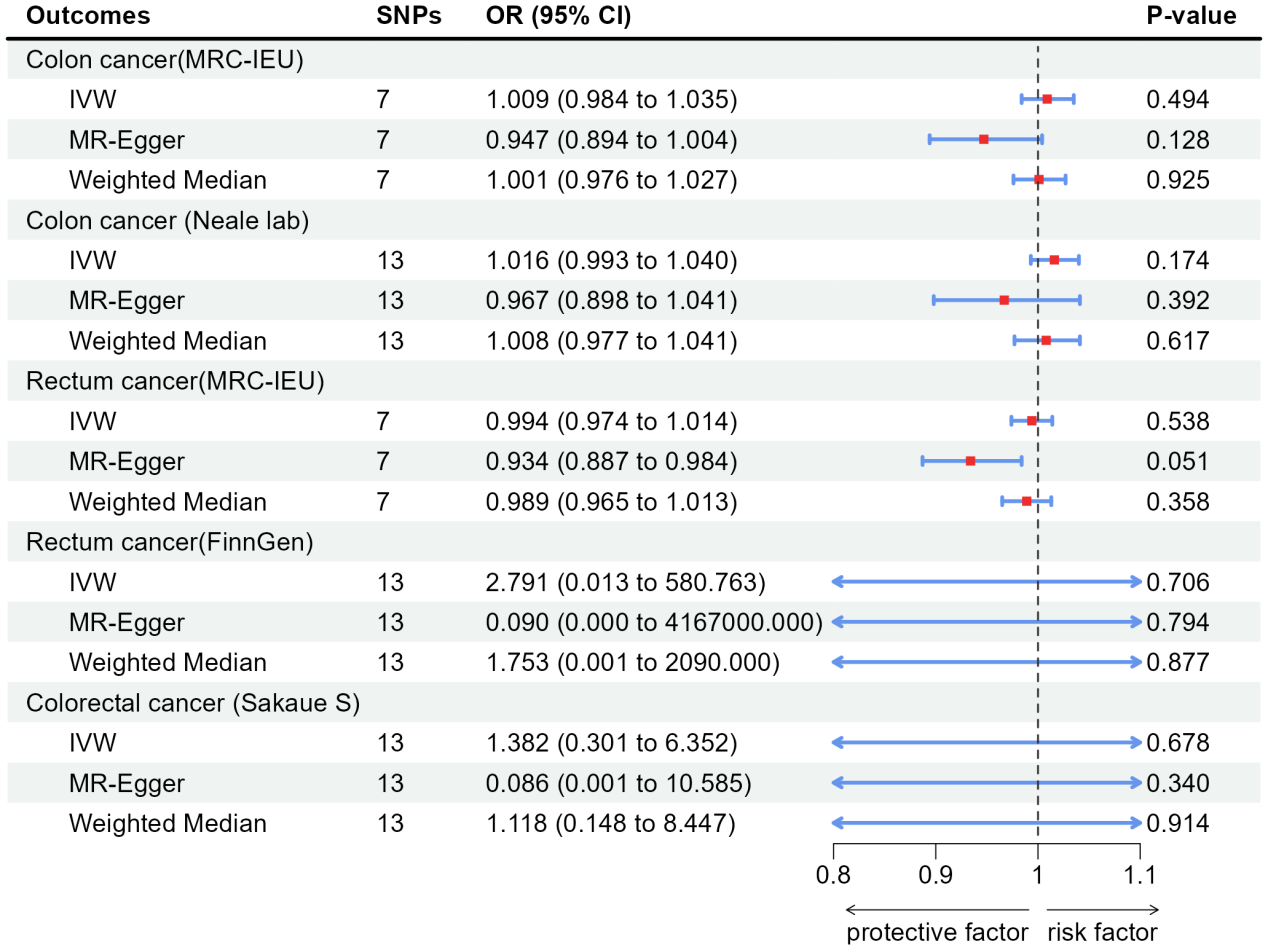
Associations of genetically predicted appendectomy with colorectal cancer by IVW, MR-Egger and Weighted Median. SNPs, single-nucleotide polymorphisms; OR, odds ratio; CI, confidence interval; IVW, inverse-variance weighted method.

According to the results of IVW, appendectomy did not increase risk of CC: Colon cancer(MRC-IEU)(OR:1.009, 95%CI:0.984-1.035, P=0.494), Colon cancer(Neale lab)(OR:1.016, 95%CI:0.993-1.040, P=0.174); Appendectomy also did not increase risk of RC: Rectum cancer(MRC-IEU)(OR:0.994, 95%CI:0.974-1.014, P=0.538), Rectum cancer(FinnGen)(OR:2.791, 95%CI:0.013-580.763, P=0.706); Appendectomy did not increase risk of CRC: Colorectal cancer (Sakaue S): (OR:1.382, 95%CI:0.301-6.352, P=0.678). Weighted Median method also showed appendectomy did not increase risk of CRC (P were all greater than 0.05). The results of heterogeneity test showed that the common effect model should be selected (P were all greater than 0.05). The results of Pleiotropy, MR-Egger and MR-PRESSO showed that there were no horizontal pleiotropy (P were all greater than 0.05). **Figure 2** showed the main results of MR analysis. Leave-one-out analysis in **Supplementary Figures 1A–E** showed that there were no SNPs which significantly affected the result. **Supplementary Figures 2A–E** and **Supplementary Figures 3A–E** showed forest plot and scatter plot of the association between appendectomy and CRC, respectively, which were also consistent with the IVW results. The funnel plot in **Supplementary Figures 4A–E** showed that the selection of SNPs was reasonable and the analysis result was robust.

#### 3.2.2 Effect of appendicitis on CRC

**Figure 3:**
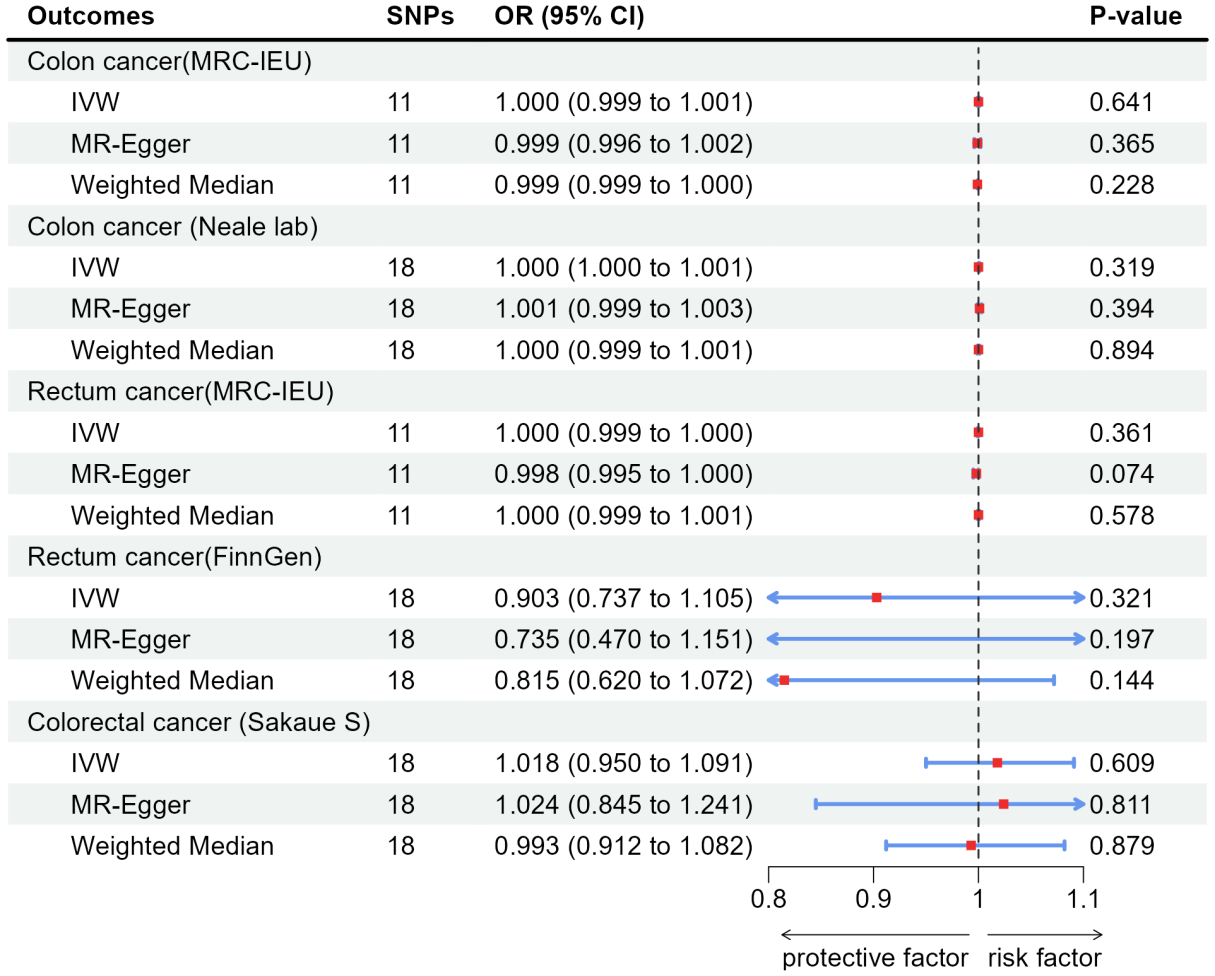
Associations of genetically predicted appendicitis with colorectal cancer by IVW, MR-Egger and Weighted Median. SNPs, single-nucleotide polymorphisms; OR, odds ratio; CI, confidence interval; IVW, inverse-variance weighted method.

According to the results of IVW, appendicitis did not increase risk of CC: Colon cancer(MRC-IEU)(OR:1.000, 95%CI:0.999-1.001, P=0.641), Colon cancer(Neale lab)(OR:1.000, 95%CI:1.000-1.001, P=0.319); Appendicitis did not increase risk of RC: Rectum cancer(MRC-IEU)(OR:1.000, 95%CI:0.999-1.000, P=0.361), Rectum cancer(FinnGen)(OR:0.903, 95%CI:0.737-1.105, P=0.321); Appendicitis also did not increase risk of CRC: Colorectal cancer (Sakaue S): (OR:1.018, 95%CI:0.950-1.091, P=0.609). Weighted Median method also showed appendicitis did not increase risk of CRC (P were all greater than 0.05). The results of heterogeneity test showed that the common effect model should be selected (P were all greater than 0.05). The results of Pleiotropy, MR-Egger and MR-PRESSO showed that there were no horizontal pleiotropy (P > 0.05). **Figure 3** showed the main results of MR analysis. Leave-one-out analysis in **Supplementary Figures 1F–J** showed that there were no SNPs which significantly affected the result. **Supplementary Figures 2F–J** and **Supplementary Figures 3F–J** showed forest plot and scatter plot of the association between appendicitis and CRC, respectively, which were also consistent with the IVW results. The funnel plot in **Supplementary Figures 4F–J** showed that the selection of SNPs was reasonable and the analysis result was robust.

### 3.3 Results of Meta-analysis

#### 3.3.1 Meta-analysis to evaluate effect of appendectomy on CRC

**Figure 4:**
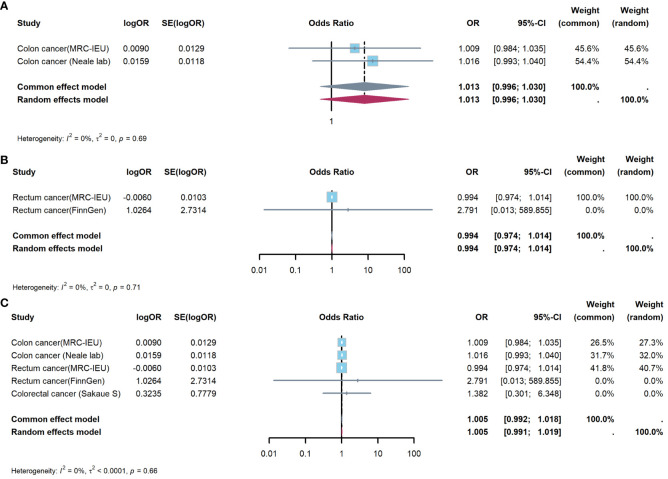
Meta-analysis of IVW results to predict associations of appendectomy with colorectal cancer. **(A)** appendectomy and colon cancer; **(B)** appendectomy and rectum cancer; **(C)** appendectomy and colorectal cancer. IVW, inverse-variance weighted method; OR, odds ratio; CI, confidence interval.

**Figure 4** showed results of Meta-analysis, which evaluated effect of appendectomy on CRC. The results of heterogeneity test showed that the common effect model should be selected (P were all greater than 0.05). **Figure 4A** showed appendectomy did not increase risk of CC(OR:1.013, 95%CI:0.996-1.030, P=0.144); **Figure 4B** showed appendectomy did not increase risk of RC(OR:0.994, 95%CI:0.974-1.014, P=0.550); **Figure 4C** showed appendectomy did not increase risk of CRC(OR:1.005, 95%CI:0.992-1.018, P=0.459).

#### 3.3.2 Meta-analysis to evaluate effect of appendicitis on CRC

**Figure 5:**
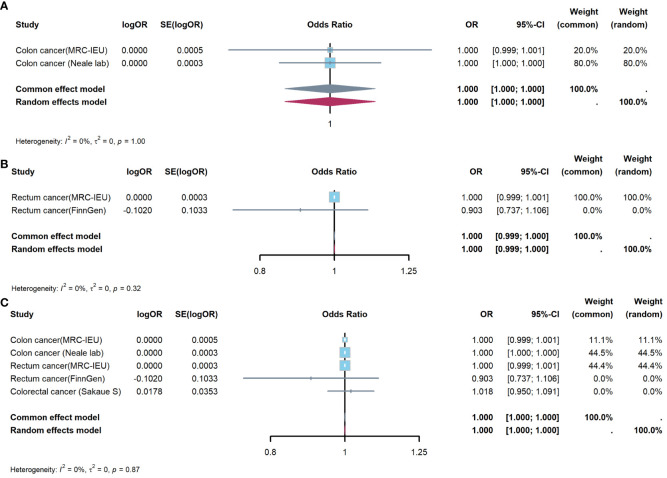
Meta-analysis of IVW results to predict associations of appendicitis with colorectal cancer. **(A)** appendicitis and colon cancer; **(B)** appendicitis and rectum cancer; **(C)** appendicitis and colorectal cancer. IVW, inverse-variance weighted method; OR, odds ratio; CI, confidence interval.

**Figure 5** showed results of Meta-analysis, which evaluated effect of appendicitis on CRC. The results of heterogeneity test showed that the common effect model should be selected (P were all greater than 0.05). **Figure 5A** showed appendicitis did not increase risk of CC(OR:1.000, 95%CI:1.000-1.000, P=1.000); **Figure 5B** showed appendicitis did not increase risk of RC(OR:1.000, 95%CI:0.999-1.000, P=0.998); **Figure 5C** showed appendicitis did not increase risk of CRC(OR:1.000, 95%CI:1.000-1.000, P=0.999).

## 4 Discussion

The incidence of appendicitis is extremely high in the world, and appendectomy is the definitive treatment of appendicitis (1, 2, 6–8). However, whether appendicitis and appendectomy increase the risk of long-term CRC has been controversial (9–14). One of the reasons for the controversy is that retrospective studies and animal experiments show opposite results, while RCTs cannot be carried out (9–14). The evidence of MR is equivalent to that of RCT, which provides a possible way to resolve this controversy (17).

We selected several GWAS summary data for CRC, and IVW results showed that appendicitis and appendectomy did not increase the risk of CRC (P were all greater than 0.05). In order to further increased the robustness of the results, we used Meta-analysis to summarize the results of IVW analysis. The results of Meta-analysis showed appendectomy (P=0.459) and appendicitis (P=0.999) did not increase the risk of CRC, which were consistent with results of IVW. Our analysis confirms previous findings from Song and Mándi et al. (21, 22).

Meta-analysis from Joseph et al. showed appendectomy was associated with a lower risk of CC (HR = 0.90, 95% CI = 0.81‐0.99) and distal CC (HR = 0.77, 95% CI = 0.65‐0.90) (13). Research results of van et al. showed appendectomy was associated with a lower risk of gastrointestinal cancer (HR 0.75, 95% CI 0.56-0.99), in particular CC (HR 0.65, 95% 0.43-0.97) (14). A possible explanation is that the appendix is ​​the germinal center of lymphoid follicles, and removing the appendix can help reduce intestinal inflammation (13, 23). It is well known that long-term chronic inflammation of gut is one of the causes of CRC; Secondly, the occurrence and progression of CRC are related to imbalance of gut microbes (13, 24). There are opportunistic pathogenic bacteria in the appendix cavity, and removal of the appendix reduces the possibility of opportunistic pathogenic bacteria entering the colorectum (24). However, other studies found that appendectomy increased the risk of CRC, and even appendicitis itself was a precursor to colorectal cancer (9–12, 25–27):results from Shi et al. showed a 73.0% increase in CRC risk among appendectomy cases throughout 20 years follow-up (SHR:1.73, 95% CI:1.49-2.01, P < 0.001) (9) and results from Lai et al. showed the odds ratio of CC incidence had a 38.5-fold increase among patients older than 40 with AA (25). The CRC risk of appendectomy and appendicitis may be due to the following reasons: first, the appendix is ​​closely related to colorectal immunity, and removal of the appendix will reduce the colorectal immune surveillance function (28, 29); Secondly, the appendix regulates the gut microbes in the colorectum, and removal of the appendix and appendicitis can aggravate gut microbes disorders (24, 30, 31). The reason for these differences may be due to the limitations of observational studies, and the evidence of this study is equivalent to RCT, which is more convincing.

Several limitations to this study deserve our attention: First, this study mainly included GWAS summary data of Europeans, and the results may not be extended to other ethnic groups; Second, subgroup analysis was not possible in this study. Stratification based on age, comorbidities, etc. may have different results; Finally, selection of SNPs is difficult. There may be confounders that cannot be identified with current knowledge.

## 5 Conclusions

Appendectomy and appendicitis do not increase the risk of colorectal cancer. More clinical trials are needed in the future to verify the causal relationships.

## Data availability statement

The original contributions presented in the study are included in the article/**Supplementary Material**, further inquiries can be directed to the corresponding author/s.

## Author contributions

WW: Data curation, Methodology, Writing – original draft. JW: Formal analysis, Funding acquisition, Writing – original draft. DY: Formal analysis, Writing – review & editing. WL: Writing – review & editing. LZ: Writing – review & editing.
