# Supplementary material for: Appendectomy and appendicitis do not increase colorectal cancer risk: evidence from Mendelian randomization
Source: Front Oncol. 2024 Jul 22;14:1414946. doi: 10.3389/fonc.2024.1414946 (PMC11298372; doi:10.3389/fonc.2024.1414946)
Supplement: Supplementary file 1 [file DataSheet_1.docx]

| Table S1. Baseline characteristics of exposure and outcomes | | | | | | | | | |
| --- | --- | --- | --- | --- | --- | --- | --- | --- | --- |
| Traits | GWAS ID | Consortium | Author | Year | Population | n case | n control | Sample Size | Number of SNPs |
| Appendicectomy | ukb-b-14944 | MRC-IEU | Ben Elsworth | 2018 | European | 55,398 | 407,535 | 462,933 | 9,851,867 |
| Appendicitis | ieu-b-4967 | UK Biobank | Hamilton F | 2021 | European | 4,604 | 481,880 | 486,484 | 12,243,521 |
| Colon cancer | ukb-b-20145 | MRC-IEU | Ben Elsworth | 2018 | European | 1,494 | 461,439 | 462,933 | 9,851,867 |
| Colon cancer | ukb-d-C18 | —— | Neale lab | 2018 | European | 2,226 | 358,968 | 361,194 | 10,833,390 |
| Rectum cancer | ukb-b-1251 | MRC-IEU | Ben Elsworth | 2018 | European | 1,470 | 461,540 | 463,010 | 9,851,867 |
| Rectum cancer | finn-b-C3_RECTUM | FinnGen | —— | 2021 | European | 1,078 | 217,714 | 218,792 | 16,380,466 |
| Colorectal cancer | ebi-a-GCST90018808 | —— | Sakaue S | 2021 | European | 6,581 | 463,421 | 470,002 | 24,182,361 |
| SNPs, single nucleotide polymorphisms. | | | | | | | | | |

| Table S2. Single nucleotide polymorphisms in the Mendelian randomization analyses of exposure and outcomes | | | | | | | | | | |
| --- | --- | --- | --- | --- | --- | --- | --- | --- | --- | --- |
| Exposure | Outcomes | SNP | Chr | EA | NEA | EAF | BETA | SE | P-value | F |
| Appendicectomy | Colon cancer (MRC-IEU) | rs7649870 | 3 | G | A | 0.51666 | -0.00433935 | 0.000675739 | 1.30E-10 | 41 |
|  |  | rs106520 | 3 | A | G | 0.260463 | -0.00422395 | 0.0007694 | 4.00E-08 | 30 |
|  |  | rs815430 | 3 | T | C | 0.752643 | -0.00436979 | 0.000784303 | 2.50E-08 | 31 |
|  |  | rs2171591 | 4 | A | G | 0.270536 | 0.00966978 | 0.000758098 | 2.90E-37 | 163 |
|  |  | rs3913815 | 4 | T | A | 0.648107 | 0.00436834 | 0.000706387 | 6.20E-10 | 38 |
|  |  | rs10829663 | 10 | G | A | 0.262555 | -0.00443209 | 0.000766008 | 7.20E-09 | 33 |
|  |  | rs2780304 | 14 | A | C | 0.287262 | 0.00443811 | 0.000751501 | 3.50E-09 | 35 |
| Appendicectomy | Rectum cancer (MRC-IEU) | rs7649870 | 3 | G | A | 0.51666 | -0.00433935 | 0.000675739 | 1.30E-10 | 41 |
|  |  | rs106520 | 3 | A | G | 0.260463 | -0.00422395 | 0.0007694 | 4.00E-08 | 30 |
|  |  | rs815430 | 3 | T | C | 0.752643 | -0.00436979 | 0.000784303 | 2.50E-08 | 31 |
|  |  | rs2171591 | 4 | A | G | 0.270536 | 0.00966978 | 0.000758098 | 2.90E-37 | 163 |
|  |  | rs3913815 | 4 | T | A | 0.648107 | 0.00436834 | 0.000706387 | 6.20E-10 | 38 |
|  |  | rs10829663 | 10 | G | A | 0.262555 | -0.00443209 | 0.000766008 | 7.20E-09 | 33 |
|  |  | rs2780304 | 14 | A | C | 0.287262 | 0.00443811 | 0.000751501 | 3.50E-09 | 35 |
| Appendicectomy | Colon cancer (Neale lab) | rs106520 | 3 | A | G | 0.260463 | -0.00422395 | 0.0007694 | 4.00E-08 | 30 |
|  |  | rs10829663 | 10 | G | A | 0.262555 | -0.00443209 | 0.000766008 | 7.20E-09 | 33 |
|  |  | rs142069498 | 20 | A | G | 0.062917 | -0.00846552 | 0.00139159 | 1.20E-09 | 37 |
|  |  | rs21525 | 7 | A | G | 0.230799 | -0.00467538 | 0.000800251 | 5.10E-09 | 34 |
|  |  | rs2171591 | 4 | A | G | 0.270536 | 0.00966978 | 0.000758098 | 2.90E-37 | 163 |
|  |  | rs2326394 | 16 | G | A | 0.838245 | 0.00646664 | 0.00091534 | 1.60E-12 | 50 |
|  |  | rs2780304 | 14 | A | C | 0.287262 | 0.00443811 | 0.000751501 | 3.50E-09 | 35 |
|  |  | rs3913815 | 4 | T | A | 0.648107 | 0.00436834 | 0.000706387 | 6.20E-10 | 38 |
|  |  | rs56259011 | 2 | T | C | 0.184706 | 0.00641125 | 0.000870315 | 1.80E-13 | 54 |
|  |  | rs72827881 | 17 | C | T | 0.045534 | -0.00965645 | 0.00162496 | 2.80E-09 | 35 |
|  |  | rs7649870 | 3 | G | A | 0.51666 | -0.00433935 | 0.000675739 | 1.30E-10 | 41 |
|  |  | rs76896900 | 4 | G | T | 0.146276 | 0.00540634 | 0.000956268 | 1.60E-08 | 32 |
|  |  | rs815430 | 3 | T | C | 0.752643 | -0.00436979 | 0.000784303 | 2.50E-08 | 31 |
| Appendicectomy | Rectum cancer (FinnGen) | rs106520 | 3 | A | G | 0.260463 | -0.00422395 | 0.0007694 | 4.00E-08 | 30 |
|  |  | rs10829663 | 10 | G | A | 0.262555 | -0.00443209 | 0.000766008 | 7.20E-09 | 33 |
|  |  | rs142069498 | 20 | A | G | 0.062917 | -0.00846552 | 0.00139159 | 1.20E-09 | 37 |
|  |  | rs21525 | 7 | A | G | 0.230799 | -0.00467538 | 0.000800251 | 5.10E-09 | 34 |
|  |  | rs2171591 | 4 | A | G | 0.270536 | 0.00966978 | 0.000758098 | 2.90E-37 | 163 |
|  |  | rs2326394 | 16 | G | A | 0.838245 | 0.00646664 | 0.00091534 | 1.60E-12 | 50 |
|  |  | rs2780304 | 14 | A | C | 0.287262 | 0.00443811 | 0.000751501 | 3.50E-09 | 35 |
|  |  | rs3913815 | 4 | T | A | 0.648107 | 0.00436834 | 0.000706387 | 6.20E-10 | 38 |
|  |  | rs56259011 | 2 | T | C | 0.184706 | 0.00641125 | 0.000870315 | 1.80E-13 | 54 |
|  |  | rs72827881 | 17 | C | T | 0.045534 | -0.00965645 | 0.00162496 | 2.80E-09 | 35 |
|  |  | rs7649870 | 3 | G | A | 0.51666 | -0.00433935 | 0.000675739 | 1.30E-10 | 41 |
|  |  | rs76896900 | 4 | G | T | 0.146276 | 0.00540634 | 0.000956268 | 1.60E-08 | 32 |
|  |  | rs815430 | 3 | T | C | 0.752643 | -0.00436979 | 0.000784303 | 2.50E-08 | 31 |
| Appendicectomy | Colorectal cancer (Sakaue S) | rs106520 | 3 | A | G | 0.260463 | -0.00422395 | 0.0007694 | 4.00E-08 | 30 |
|  |  | rs10829663 | 10 | G | A | 0.262555 | -0.00443209 | 0.000766008 | 7.20E-09 | 33 |
|  |  | rs142069498 | 20 | A | G | 0.062917 | -0.00846552 | 0.00139159 | 1.20E-09 | 37 |
|  |  | rs21525 | 7 | A | G | 0.230799 | -0.00467538 | 0.000800251 | 5.10E-09 | 34 |
|  |  | rs2171591 | 4 | A | G | 0.270536 | 0.00966978 | 0.000758098 | 2.90E-37 | 163 |
|  |  | rs2326394 | 16 | G | A | 0.838245 | 0.00646664 | 0.00091534 | 1.60E-12 | 50 |
|  |  | rs2780304 | 14 | A | C | 0.287262 | 0.00443811 | 0.000751501 | 3.50E-09 | 35 |
|  |  | rs3913815 | 4 | T | A | 0.648107 | 0.00436834 | 0.000706387 | 6.20E-10 | 38 |
|  |  | rs56259011 | 2 | T | C | 0.184706 | 0.00641125 | 0.000870315 | 1.80E-13 | 54 |
|  |  | rs72827881 | 17 | C | T | 0.045534 | -0.00965645 | 0.00162496 | 2.80E-09 | 35 |
|  |  | rs7649870 | 3 | G | A | 0.51666 | -0.00433935 | 0.000675739 | 1.30E-10 | 41 |
|  |  | rs76896900 | 4 | G | T | 0.146276 | 0.00540634 | 0.000956268 | 1.60E-08 | 32 |
|  |  | rs815430 | 3 | T | C | 0.752643 | -0.00436979 | 0.000784303 | 2.50E-08 | 31 |
| Appendicitis | Colon cancer (MRC-IEU) | rs1239688 | 13 | A | C | 0.535011 | -0.0992844 | 0.0215089 | 3.91E-06 | 21 |
|  |  | rs13121924 | 4 | G | A | 0.637526 | -0.204143 | 0.0223055 | 5.58E-20 | 84 |
|  |  | rs2387620 | 8 | C | T | 0.324153 | -0.102014 | 0.0229737 | 8.98E-06 | 20 |
|  |  | rs34882722 | 14 | T | C | 0.655618 | -0.100827 | 0.0225864 | 8.04E-06 | 20 |
|  |  | rs56090943 | 3 | T | C | 0.365459 | 0.0999636 | 0.0223137 | 7.47E-06 | 20 |
|  |  | rs6021661 | 20 | G | A | 0.522723 | 0.0985535 | 0.0215695 | 4.90E-06 | 21 |
|  |  | rs6601903 | 10 | A | G | 0.268212 | 0.107012 | 0.0241987 | 9.77E-06 | 20 |
|  |  | rs7003854 | 8 | A | G | 0.34335 | 0.109265 | 0.0228118 | 1.67E-06 | 23 |
|  |  | rs73391926 | 22 | C | T | 0.370901 | -0.0989705 | 0.0223146 | 9.20E-06 | 20 |
|  |  | rs760608 | 6 | A | G | 0.737437 | 0.127105 | 0.0244564 | 2.02E-07 | 27 |
|  |  | rs9994167 | 4 | T | A | 0.64823 | 0.0992352 | 0.0224516 | 9.87E-06 | 20 |
| Appendicitis | Rectum cancer (MRC-IEU) | rs1239688 | 13 | A | C | 0.535011 | -0.0992844 | 0.0215089 | 3.91E-06 | 21 |
|  |  | rs13121924 | 4 | G | A | 0.637526 | -0.204143 | 0.0223055 | 5.58E-20 | 84 |
|  |  | rs2387620 | 8 | C | T | 0.324153 | -0.102014 | 0.0229737 | 8.98E-06 | 20 |
|  |  | rs34882722 | 14 | T | C | 0.655618 | -0.100827 | 0.0225864 | 8.04E-06 | 20 |
|  |  | rs56090943 | 3 | T | C | 0.365459 | 0.0999636 | 0.0223137 | 7.47E-06 | 20 |
|  |  | rs6021661 | 20 | G | A | 0.522723 | 0.0985535 | 0.0215695 | 4.90E-06 | 21 |
|  |  | rs6601903 | 10 | A | G | 0.268212 | 0.107012 | 0.0241987 | 9.77E-06 | 20 |
|  |  | rs7003854 | 8 | A | G | 0.34335 | 0.109265 | 0.0228118 | 1.67E-06 | 23 |
|  |  | rs73391926 | 22 | C | T | 0.370901 | -0.0989705 | 0.0223146 | 9.20E-06 | 20 |
|  |  | rs760608 | 6 | A | G | 0.737437 | 0.127105 | 0.0244564 | 2.02E-07 | 27 |
|  |  | rs9994167 | 4 | T | A | 0.64823 | 0.0992352 | 0.0224516 | 9.87E-06 | 20 |
| Appendicitis | Colon cancer (Neale lab) | rs117131647 | 16 | G | A | 0.0210353 | 0.356769 | 0.0760498 | 2.72E-06 | 22 |
|  |  | rs117586216 | 10 | C | T | 0.0446926 | -0.233636 | 0.0516872 | 6.18E-06 | 20 |
|  |  | rs118188003 | 16 | G | C | 0.0144869 | -0.423946 | 0.0909931 | 3.18E-06 | 22 |
|  |  | rs1239688 | 13 | A | C | 0.535011 | -0.0992844 | 0.0215089 | 3.91E-06 | 21 |
|  |  | rs13121924 | 4 | G | A | 0.637526 | -0.204143 | 0.0223055 | 5.58E-20 | 84 |
|  |  | rs2181572 | 6 | C | A | 0.0628163 | -0.225943 | 0.0457392 | 7.82E-07 | 24 |
|  |  | rs2387620 | 8 | C | T | 0.324153 | -0.102014 | 0.0229737 | 8.98E-06 | 20 |
|  |  | rs34882722 | 14 | T | C | 0.655618 | -0.100827 | 0.0225864 | 8.04E-06 | 20 |
|  |  | rs56090943 | 3 | T | C | 0.365459 | 0.0999636 | 0.0223137 | 7.47E-06 | 20 |
|  |  | rs57682625 | 16 | T | C | 0.0745052 | -0.185623 | 0.0412465 | 6.78E-06 | 20 |
|  |  | rs6021661 | 20 | G | A | 0.522723 | 0.0985535 | 0.0215695 | 4.90E-06 | 21 |
|  |  | rs6601903 | 10 | A | G | 0.268212 | 0.107012 | 0.0241987 | 9.77E-06 | 20 |
|  |  | rs7003854 | 8 | A | G | 0.34335 | 0.109265 | 0.0228118 | 1.67E-06 | 23 |
|  |  | rs73391926 | 22 | C | T | 0.370901 | -0.0989705 | 0.0223146 | 9.20E-06 | 20 |
|  |  | rs760608 | 6 | A | G | 0.737437 | 0.127105 | 0.0244564 | 2.02E-07 | 27 |
|  |  | rs77377460 | 20 | T | C | 0.0190862 | 0.373703 | 0.0779911 | 1.65E-06 | 23 |
|  |  | rs864705 | 3 | A | G | 0.0121685 | 0.465642 | 0.0992927 | 2.74E-06 | 22 |
|  |  | rs9994167 | 4 | T | A | 0.64823 | 0.0992352 | 0.0224516 | 9.87E-06 | 20 |
| Appendicitis | Rectum cancer (FinnGen) | rs117131647 | 16 | G | A | 0.0210353 | 0.356769 | 0.0760498 | 2.72E-06 | 22 |
|  |  | rs117586216 | 10 | C | T | 0.0446926 | -0.233636 | 0.0516872 | 6.18E-06 | 20 |
|  |  | rs118188003 | 16 | G | C | 0.0144869 | -0.423946 | 0.0909931 | 3.18E-06 | 22 |
|  |  | rs1239688 | 13 | A | C | 0.535011 | -0.0992844 | 0.0215089 | 3.91E-06 | 21 |
|  |  | rs13121924 | 4 | G | A | 0.637526 | -0.204143 | 0.0223055 | 5.58E-20 | 84 |
|  |  | rs2181572 | 6 | C | A | 0.0628163 | -0.225943 | 0.0457392 | 7.82E-07 | 24 |
|  |  | rs2387620 | 8 | C | T | 0.324153 | -0.102014 | 0.0229737 | 8.98E-06 | 20 |
|  |  | rs34882722 | 14 | T | C | 0.655618 | -0.100827 | 0.0225864 | 8.04E-06 | 20 |
|  |  | rs56090943 | 3 | T | C | 0.365459 | 0.0999636 | 0.0223137 | 7.47E-06 | 20 |
|  |  | rs57682625 | 16 | T | C | 0.0745052 | -0.185623 | 0.0412465 | 6.78E-06 | 20 |
|  |  | rs6021661 | 20 | G | A | 0.522723 | 0.0985535 | 0.0215695 | 4.90E-06 | 21 |
|  |  | rs6601903 | 10 | A | G | 0.268212 | 0.107012 | 0.0241987 | 9.77E-06 | 20 |
|  |  | rs7003854 | 8 | A | G | 0.34335 | 0.109265 | 0.0228118 | 1.67E-06 | 23 |
|  |  | rs73391926 | 22 | C | T | 0.370901 | -0.0989705 | 0.0223146 | 9.20E-06 | 20 |
|  |  | rs760608 | 6 | A | G | 0.737437 | 0.127105 | 0.0244564 | 2.02E-07 | 27 |
|  |  | rs77377460 | 20 | T | C | 0.0190862 | 0.373703 | 0.0779911 | 1.65E-06 | 23 |
|  |  | rs864705 | 3 | A | G | 0.0121685 | 0.465642 | 0.0992927 | 2.74E-06 | 22 |
|  |  | rs9994167 | 4 | T | A | 0.64823 | 0.0992352 | 0.0224516 | 9.87E-06 | 20 |
| Appendicitis | Colorectal cancer (Sakaue S) | rs117131647 | 16 | G | A | 0.0210353 | 0.356769 | 0.0760498 | 2.72E-06 | 22 |
|  |  | rs117586216 | 10 | C | T | 0.0446926 | -0.233636 | 0.0516872 | 6.18E-06 | 20 |
|  |  | rs118188003 | 16 | G | C | 0.0144869 | -0.423946 | 0.0909931 | 3.18E-06 | 22 |
|  |  | rs1239688 | 13 | A | C | 0.535011 | -0.0992844 | 0.0215089 | 3.91E-06 | 21 |
|  |  | rs13121924 | 4 | G | A | 0.637526 | -0.204143 | 0.0223055 | 5.58E-20 | 84 |
|  |  | rs2181572 | 6 | C | A | 0.0628163 | -0.225943 | 0.0457392 | 7.82E-07 | 24 |
|  |  | rs2387620 | 8 | C | T | 0.324153 | -0.102014 | 0.0229737 | 8.98E-06 | 20 |
|  |  | rs34882722 | 14 | T | C | 0.655618 | -0.100827 | 0.0225864 | 8.04E-06 | 20 |
|  |  | rs56090943 | 3 | T | C | 0.365459 | 0.0999636 | 0.0223137 | 7.47E-06 | 20 |
|  |  | rs57682625 | 16 | T | C | 0.0745052 | -0.185623 | 0.0412465 | 6.78E-06 | 20 |
|  |  | rs6021661 | 20 | G | A | 0.522723 | 0.0985535 | 0.0215695 | 4.90E-06 | 21 |
|  |  | rs6601903 | 10 | A | G | 0.268212 | 0.107012 | 0.0241987 | 9.77E-06 | 20 |
|  |  | rs7003854 | 8 | A | G | 0.34335 | 0.109265 | 0.0228118 | 1.67E-06 | 23 |
|  |  | rs73391926 | 22 | C | T | 0.370901 | -0.0989705 | 0.0223146 | 9.20E-06 | 20 |
|  |  | rs760608 | 6 | A | G | 0.737437 | 0.127105 | 0.0244564 | 2.02E-07 | 27 |
|  |  | rs77377460 | 20 | T | C | 0.0190862 | 0.373703 | 0.0779911 | 1.65E-06 | 23 |
|  |  | rs864705 | 3 | A | G | 0.0121685 | 0.465642 | 0.0992927 | 2.74E-06 | 22 |
|  |  | rs9994167 | 4 | T | A | 0.64823 | 0.0992352 | 0.0224516 | 9.87E-06 | 20 |
| SNP, single nucleotide polymorphism; Chr, chromosome; EA, effect allele; NEA, non effect allele; EAF, effect allele frequency; SE, standard error. | | | | | | | | | | |


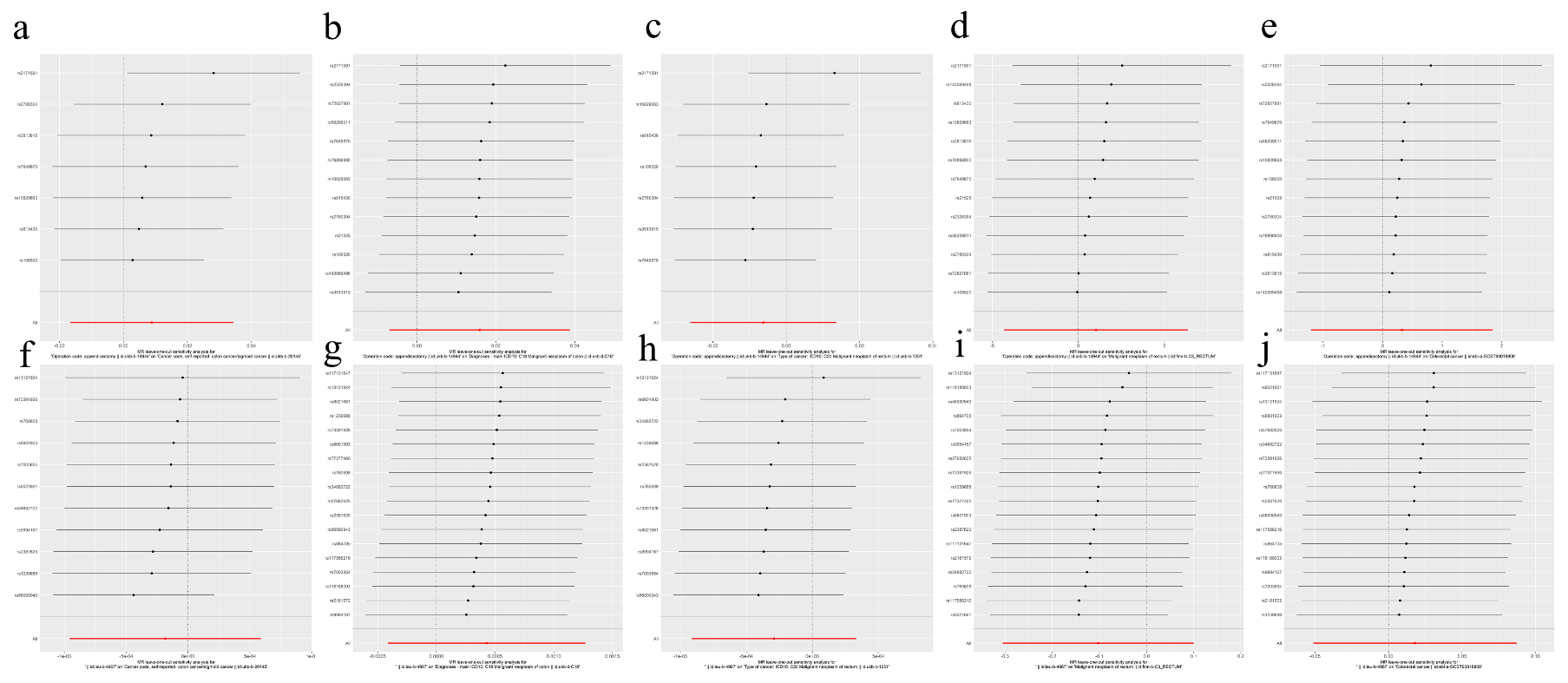


Supplementary Figure 1. Leave-one-out results of the association between exposure factors and outcomes. (a)-(e): Effects of appendicectomy on (a) Colon cancer (MRC-IEU); (b) Colon cancer (Neale lab); (c) Rectum cancer (MRC-IEU); (d) Rectum cancer (FinnGen); (e) Colorectal cancer (Sakaue S); (f)-(j): Effects of appendicitis on (f) Colon cancer (MRC-IEU); (g) Colon cancer (Neale lab); (h) Rectum cancer (MRC-IEU); (i) Rectum cancer (FinnGen); (j) Colorectal cancer (Sakaue S).


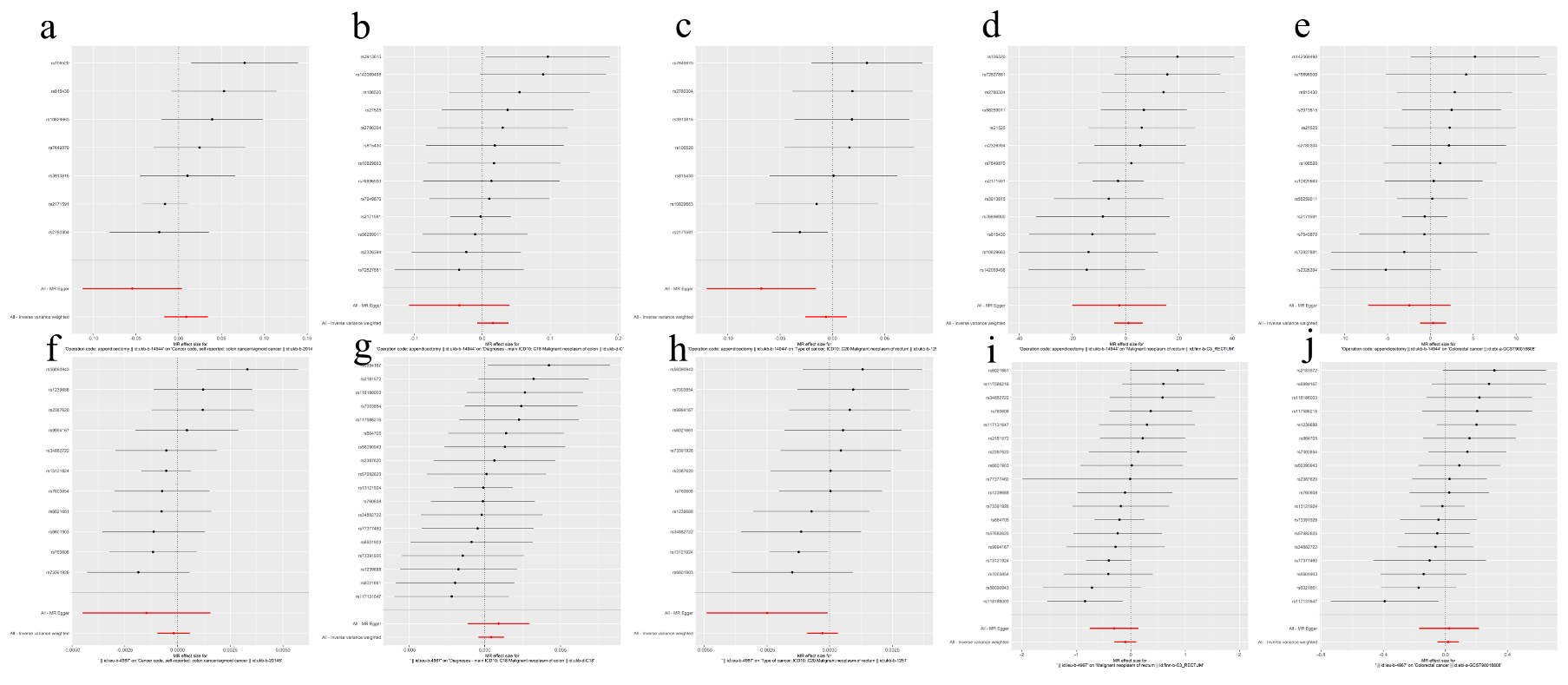


Supplementary Figure 2. Forest plot of the association between exposure factors and outcomes. (a)-(e): Effects of appendicectomy on (a) Colon cancer (MRC-IEU);

(b) Colon cancer (Neale lab); (c) Rectum cancer (MRC-IEU); (d) Rectum cancer (FinnGen); (e) Colorectal cancer (Sakaue S); (f)-(j): Effects of appendicitis on (f) Colon cancer (MRC-IEU); (g) Colon cancer (Neale lab); (h) Rectum cancer (MRC-IEU); (i) Rectum cancer (FinnGen); (j) Colorectal cancer (Sakaue S).


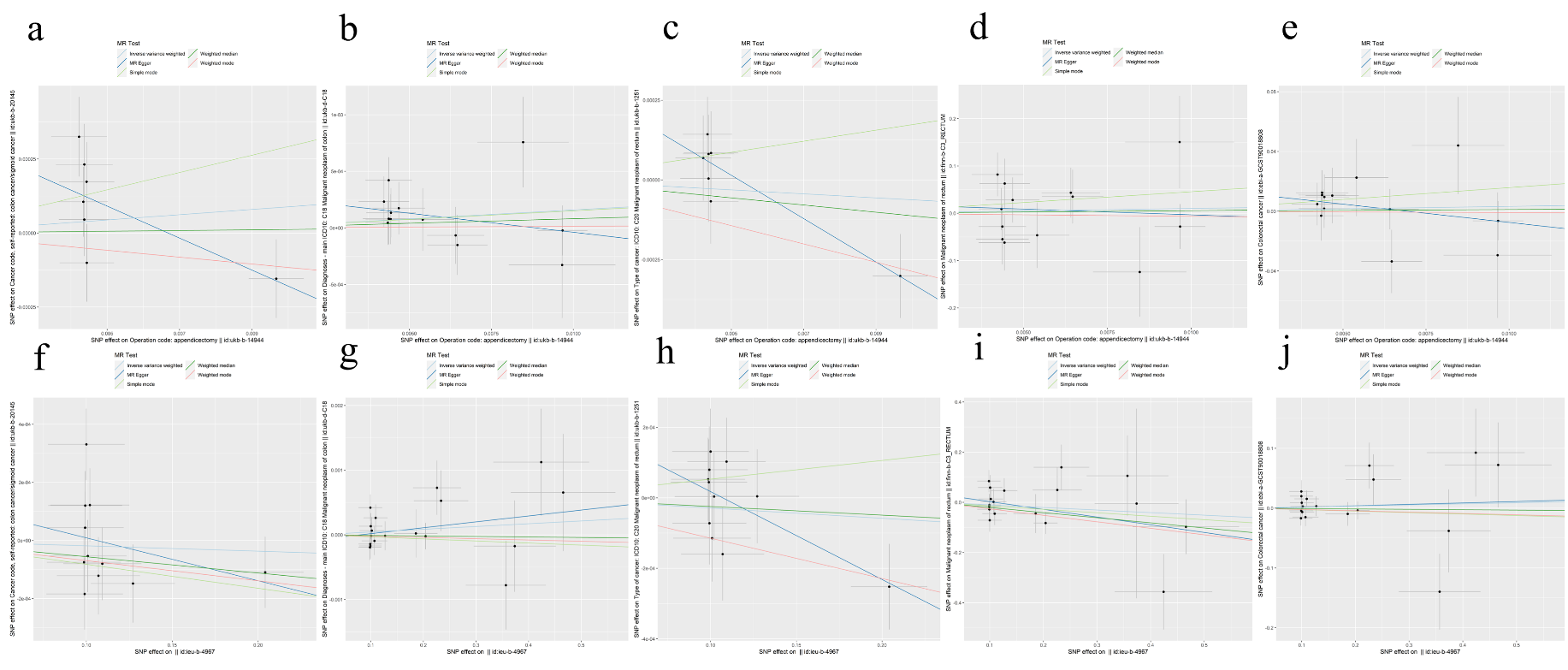


Supplementary Figure 3. Scatter plot of the association between exposure factors and outcomes. (a)-(e): Effects of appendicectomy on (a) Colon cancer (MRC-IEU);

(b) Colon cancer (Neale lab); (c) Rectum cancer (MRC-IEU); (d) Rectum cancer (FinnGen); (e) Colorectal cancer (Sakaue S); (f)-(j): Effects of appendicitis on (f) Colon cancer (MRC-IEU); (g) Colon cancer (Neale lab); (h) Rectum cancer (MRC-IEU); (i) Rectum cancer (FinnGen); (j) Colorectal cancer (Sakaue S).


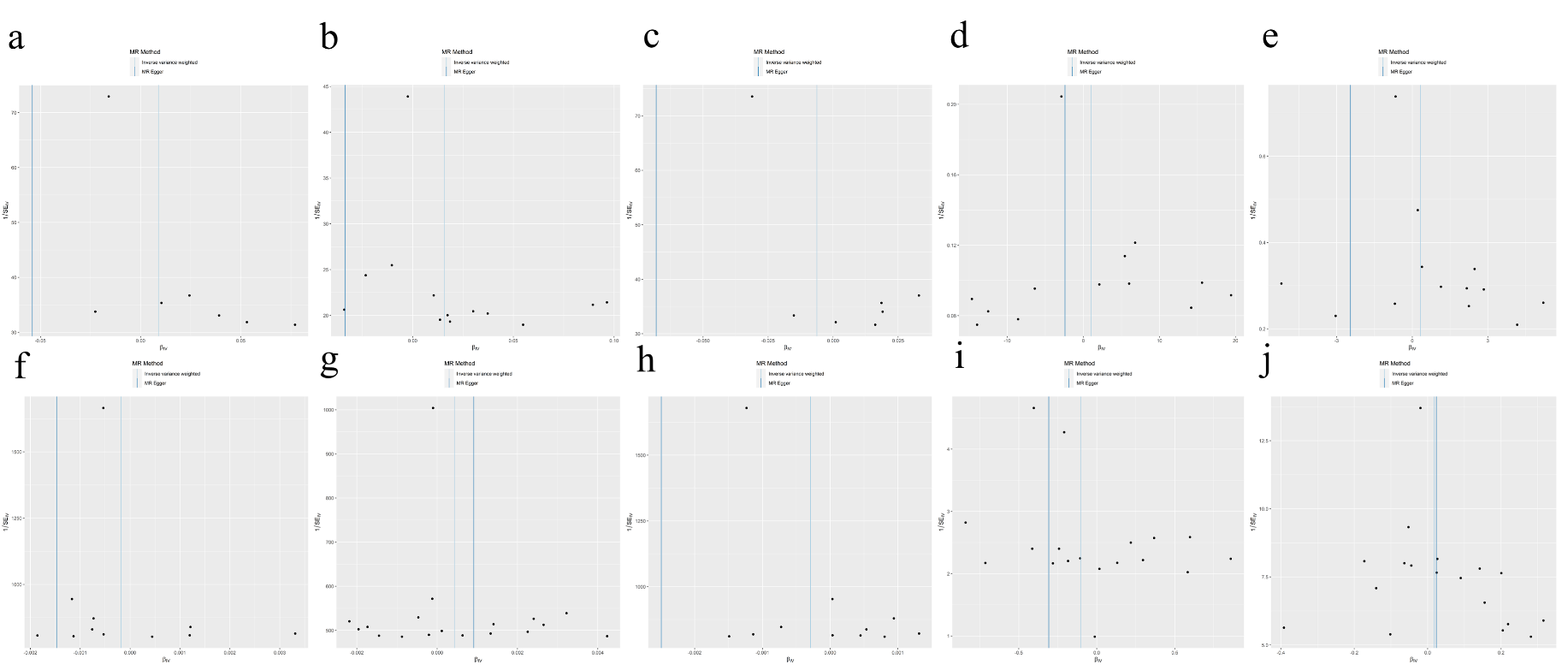


Supplementary Figure 4. Funnel plot of the association between exposure factors and outcomes. (a)-(e): Effects of appendicectomy on (a) Colon cancer (MRC-IEU);

(b) Colon cancer (Neale lab); (c) Rectum cancer (MRC-IEU); (d) Rectum cancer (FinnGen); (e) Colorectal cancer (Sakaue S); (f)-(j): Effects of appendicitis on (f) Colon cancer (MRC-IEU); (g) Colon cancer (Neale lab); (h) Rectum cancer (MRC-IEU); (i) Rectum cancer (FinnGen); (j) Colorectal cancer (Sakaue S).
